# High-Throughput PET/CT Imaging Using a Multiple-Mouse Imaging System

**DOI:** 10.2967/jnumed.119.228692

**Published:** 2020-02

**Authors:** Hannah E. Greenwood, Zoltan Nyitrai, Gabor Mocsai, Sandor Hobor, Timothy H. Witney

**Affiliations:** 1 Centre for Advanced Biomedical Imaging, Division of Medicine, University College London, London, United Kingdom; 2 Department of Imaging Chemistry and Biology, School of Biomedical Engineering and Imaging Sciences, King’s College London, London, United Kingdom; and; 3 Mediso Medical Imaging Systems, Budapest, Hungary

**Keywords:** high-throughput, mouse hotel, micro PET/CT, ^18^F-FDG, economic

## Abstract

A considerable limitation of current small-animal PET/CT imaging is the low throughput of acquisitions. Consequently, to sufficiently power a study, high costs accumulate. Together with a commercial scanner manufacturer, we developed a 4-bed mouse “hotel” to simultaneously image up to 4 mice, thereby reducing costs and maximizing the efficiency of radiotracer use when compared with scans performed with a single mouse bed. **Methods:** For physiologic evaluation of the mouse hotel, temperature and anesthesia were tested for uniformity in conjunction with ^18^F-FDG PET/CT imaging of mini image-quality phantoms designed to fit the new imaging system. After reconstruction, National Electrical Manufacturers Association NU-4 tests examined uniformity, recovery coefficients, and spillover ratios. To evaluate the mouse hotel under standard in vivo imaging conditions, 4 mice were simultaneously scanned by dynamic ^18^F-FDG PET/CT over 60 min, and quantified images were compared with those acquired using a single mouse bed. **Results:** The mouse hotel maintained a constant temperature of 36.8°C ± 0.4°C, with anesthesia distributed evenly to each nose cone (2.9 ± 0.1 L/min). The National Electrical Manufacturers Association tests revealed values within tolerable limits for uniformity, for recovery coefficients in rods larger than 2 mm, and for spillover ratios in the nonradioactive water- and air-filled chambers. There was low variability in radiotracer uptake in all major organs for the mouse hotel versus the single mouse bed. **Conclusion:** Analysis of images acquired using the mouse hotel confirmed its utility to increase the throughput of small-animal PET imaging without considerable loss of image quality or quantitative precision. In comparison to a single mouse bed, the cost and time associated with each scan were substantially reduced.

As a noninvasive imaging tool, PET is used in preclinical research across multidisciplinary areas of work for whole-body, dynamic examination of biochemical processes under normal and pathophysiologic conditions (1–4). As a translational tool, preclinical PET has enabled the development of many different radiotracers currently in clinical use. For example, the development of radiotracers targeting prostate-specific membrane antigen for human prostate cancer imaging, theranostic approaches, and the ability to track therapeutic cells in vivo all stem from their thorough evaluation in rodent models (5–7).

A considerable limitation of current small-animal PET/CT imaging is the low throughput of image acquisitions. Single-mouse-bed imaging becomes particularly restrictive when radioactive isotopes with short half-lives, such as ^11^C and ^18^F, or complex dynamic imaging studies are used. Subsequently, to sufficiently power a study, high costs accumulate. For many research groups, these high imaging costs present a barrier to widespread preclinical adoption of PET and may restrict the frequency of radioactive preparations available to others who have invested in this imaging modality. A potential solution to this economic problem is to scan multiple animals simultaneously. Several commercially available preclinical scanners have adequate axial length and diameter to achieve multianimal imaging, and as a result, many user-designed multianimal holders have entered routine application (8–10). Often, however, these beds lack the thorough characterization required for the production of reproducible and quantifiable PET data, with animal heating and monitoring capabilities frequently omitted from the bed design. It is well recognized that temperature and anesthesia can greatly affect the biodistribution of injected radiotracers, and so maintaining control of these variables is essential to maintain reproducibility across subjects and sites (11). It is also important to consider the impact on image quality and quantitative accuracy of scanning multiple animals in the same field of view. The presence of more than one concentrated source of radioactivity may negatively affect attenuation, increase the singles and randoms rates, increase the number of scatter events, increase the detector and system dead time, and reduce resolution and sensitivity as subjects are placed away from the center of the field of view.

To examine and overcome the low throughput of conventional PET imaging, we developed and validated a 4-bed mouse “hotel” in collaboration with Mediso Imaging Systems. The mouse hotel was designed to deliver an even distribution of anesthesia to each nose cone and maintain animals at 37°C. Heat and temperature tests were performed, and the National Electrical Manufacturers Association (NEMA) NU-4 2008 standard protocol for small-animal PET scans was used to assess the image quality of phantom PET scans. We then investigated whether the mouse hotel negatively affects the quality of in vivo dynamic PET images simultaneously acquired with 4 mice. After injection of ^18^F-FDG into female BALB/c mice, images were obtained and compared with those acquired using a single mouse bed.

## MATERIALS AND METHODS

### Design and Development of Mouse Hotel

**FIGURE 1.**
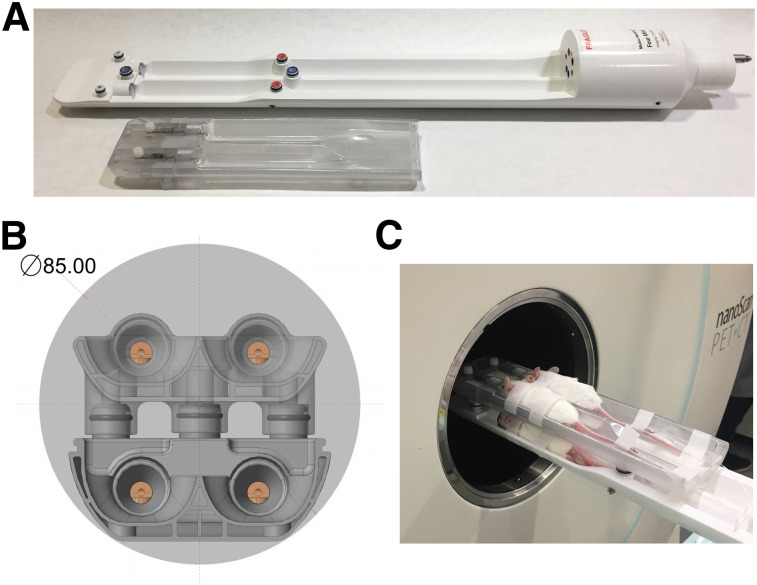
Design of mouse hotel. (A) Modular design with removable top bed allows 1–4 mice weighing 50 g to be simultaneously imaged. (B) Cross-section of bed within PET field of view (large light gray circle). Four individual nose cones are visible, with air-filled chamber located under each mouse bed also in view. (C) Photograph of 4 female BALB/c mice within mouse hotel.

We designed the mouse hotel for use in a nanoScan PET/CT system (Mediso Medical Imaging Systems) with the system’s manufacturers (Fig. 1A). The mouse hotel is made of high-temperature–resistant plastic, has 4 holding chambers with individual nose cones for anesthesia delivery (Fig. 1B), contains chambers to allow the flow of heated air to all 4 beds (with each mouse placed equidistant from the center of the field of view), and allows simultaneous imaging of up to 4 mice within the same single field of view (Fig. 1C). The modular design allowed removal of the top bed layer when only 1–2 mice are required for imaging, such as when increasing the group size to 5 or more animals.

### NEMA Mini Image-Quality Phantom Studies

**FIGURE 2.**
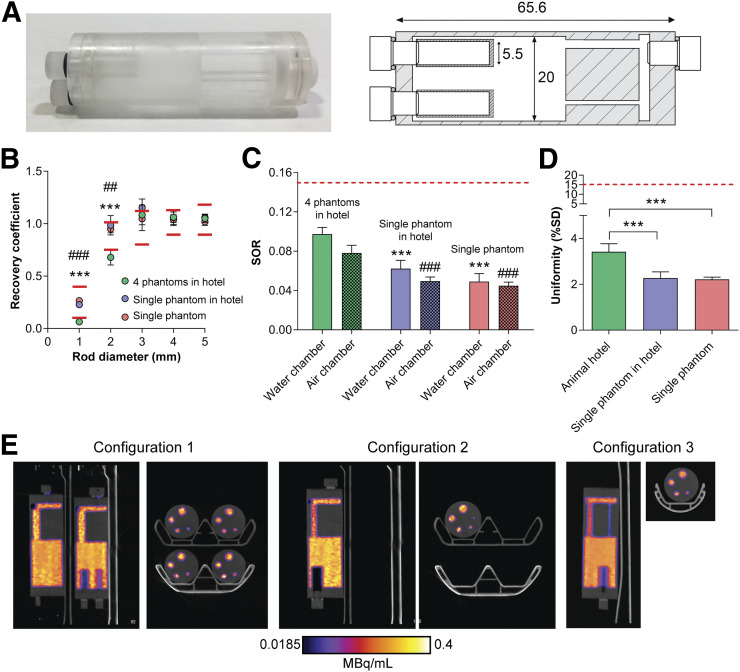
(A) Photograph and diagram of mini image-quality NEMA NU-4 phantom, with dimensions shown in millimeters. (B–D) RC of 1- to 5-mm rods (B), SORs (C), and uniformity values (D) for phantoms imaged using mouse hotel. (E) Representative sagittal and axial PET images of 0- to 20-min summed activity. Solid and dashed red lines represent tolerable limits set by NEMA NU-4. In B: ***P < 0.001 for configuration 1 vs. 2; ###*P* < 0.001 for configuration 1 vs. 3. In C: ***P < 0.001 for configuration 1 water SOR vs. configurations 2 and 3; ###*P* < 0.001 for configuration 1 air SOR vs. configurations 2 and 3. ##*P* < 0.01 for configuration 1 vs 3. ***P < 0.001.

Cylindric mouse-sized phantoms were designed by Mediso Medical Imaging Systems specifically for use in the mouse hotel (Fig. 2A). Each phantom, made from acrylic glass, consisted of 3 parts: a large, uniform compartment; a solid region with 5 fillable rods 1, 2, 3, 4, and 5 mm in diameter; and a nonradioactive region containing water- and air-filled chambers, designed to replicate the conditions of an in vivo PET/CT scan. In comparison to the standard NEMA image-quality phantom, the diameter of the mini phantom was reduced from 30 to 20 mm, which reduced the phantom volume from 20 to 10 mL. In addition, the spillover-ratio chambers were reduced to a diameter of 5.5 mm in the mini phantom versus 8 mm in the standard phantom. The image-quality program used to determine scanner performance (Mediso) was modified on the basis of NEMA conversions for these changes to meet the NEMA standards.

Following the guidelines suggested by NEMA NU-4, we filled each phantom with 3.7 MBq of ^18^F-FDG in 10 mL of phosphate-buffered saline (Thermo Scientific), decay-corrected to the start of acquisition. Clinical-grade ^18^F-FDG was obtained from PETNET solutions. The solutions in the phantoms were thoroughly mixed and bubbles carefully removed before the phantoms were placed onto the imaging bed. The phantoms were imaged using different arrangements within the bed. For multiple-phantom scans, 4 phantoms were placed in the mouse hotel at the same time (configuration 1). For single-phantom scans, 1 phantom was placed into each bed location within the hotel and imaged individually (configuration 2). Additionally, a single phantom was imaged using a single mouse bed for comparison (configuration 3).

Four phantoms were imaged in the mouse hotel following the exact specification suggested by the PET manufacturer for single-mouse imaging. A dynamic PET acquisition was performed on a nanoScan PET/CT system over 20 min followed by CT (480 projections, 50-kVp tube voltage, 600 μA, 300-ms exposure time, 1:4 binning, and helical acquisition). Whole-body Tera-Tomo (Mediso) 3-dimensional reconstruction with 4 iterations and 6 subsets was performed (1–5 coincidence mode) using an isotropic voxel size of 0.3 mm^3^. Images were corrected for attenuation, scatter, and decay. A gaussian filter of 0.7 was added to the reconstructed PET images using VivoQuant software (version 2.5; Invicro Ltd.).

To optimize the manufacturer’s suggested scanner and reconstruction parameters, the tube voltage for CT imaging was increased to 70 kVp, reducing the current to 310 μA. Additionally, whole-body Tera-Tomo 3-dimensional reconstruction with 10 iterations and 6 subsets was performed (1–5 coincidence mode) using an isotropic voxel size of 0.3 mm^3^ following an iterative process to quantitatively improve bed performance. Images were corrected for attenuation, scatter, and decay. A gaussian filter of 0.7 was added to the reconstructed PET images using VivoQuant software (version 2.5).

### NEMA NU-4 2008 Tests

After reconstruction, NEMA NU-4 2008 tests were performed to evaluate the effect of multiple subjects on scanner performance. Image noise was expressed as percentage SD by selecting a large field of view (75% of the active diameter) in the center of the fillable region of the phantom. Activity recovery coefficients (RCs) in the 5 fillable rods were calculated from the maximum detected activity in each rod, divided by the mean total phantom activity concentration. To evaluate scatter correction, spillover ratios (SORs) in the nonradioactive water- and air-filled chambers were measured as the activity detected in these regions, divided by the mean total phantom activity concentration. Data were exported and analyzed in Prism (version 8.0; GraphPad).

### Temperature and Anesthesia Tests

The air flow temperature delivered to the bed was set at 38°C and allowed to plateau for 5 min before temperature readings were taken using a thermal camera (FLIR systems AB-E60). Each animal holding bed within the hotel was measured individually at multiple positions. The temperature of the bottom bed layer was assessed after removal of the top layer. To measure anesthesia delivery, a flowmeter (Dwyer RMA-26–68V) was attached to each individual anesthesia nose cone.

### PET/CT Animal Imaging Studies

All animal experiments were performed in accordance with the U.K. Home Office Animal (scientific procedures) Act of 1986. The PET acquisition was performed on a nanoScan PET/CT system. Female BALB/c mice aged 9–12 wk (Charles River Laboratories) were fasted for 24 h before image acquisition. The mice were anesthetized with 2.5% isoflurane in oxygen, and a tail vein cannula was inserted. A bolus of 3.7 MBq of ^18^F-FDG was injected in approximately 100 μL of phosphate-buffered saline, and a 60-min dynamic acquisition began immediately. For attenuation correction and anatomic reference, CT images were acquired after PET imaging (480 projections, 70-kVp tube voltage, 300-ms exposure time, 1:4 binning, and helical acquisition). Animals received 2.5% isoflurane in oxygen throughout the scan and were maintained at 37°C by the airflow-heated bed. Breathing rate and body temperature were visually monitored for all animals throughout the imaging procedure.

The acquired data were sorted into 19 time frames of 4 × 15 s, 4 × 60 s, and 11 × 300 s for image reconstruction (whole-body Tera-Tomo 3-dimensional reconstruction with 10 iterations and 6 subsets; 400–600 keV; 0.4 mm^3^ voxel size). VivoQuant software (version 2.5) was used to analyze the reconstructed images. Regions of interest were drawn manually using CT images and 30- to 60-min summed dynamic PET images as a reference. Time–activity curves were generated using count densities normalized to the injected activity, and the area under the time–activity curve was generated.

### Statistics

All data were expressed as mean ± SD. Statistical significance was determined using either a 2-tailed *t* test or ANOVA followed by *t* test multiple-comparison correction (Tukey method; Prism, version 8.0).

## RESULTS

### Phantom Studies

Quantitative analysis of the phantom images was performed using NEMA NU-4 tests after reconstruction, with the tolerable limits set by NEMA shown in Supplemental Table 1 (supplemental materials are available at http://jnm.snmjournals.org). Initially, phantom images were reconstructed using those parameters recommended by the manufacturer for standard scans on a single animal. The NEMA NU-4 test results using these standard parameters are shown in Supplemental Figure 1. Subsequently, these reconstruction parameters were optimized to improve image quality. In addition, the x-ray tube voltage was increased from 50 to 70 kVP, which had minimal effect on absorbed dose (CT dose index of 3.8 vs. 3.7 cGy, respectively, for a 100-mm field of view). The RCs measured in the 5 fillable rods with diameters of 1–5 mm were used to evaluate the spatial resolution of images acquired using the mouse hotel and were compared with a single mouse bed. Reduced spatial resolution was evident in phantoms imaged using the mouse hotel when the RCs of the 1- and 2-mm rods were examined, with values falling outside tolerable limits suggested by the manufacturer’s guidelines (0.06 ± 0.01 and 0.68 ± 0.07, respectively). Spatial resolution, however, was not affected in the larger rods (1.08 ± 0.09, 1.06 ± 0.05, and 1.05 ± 0.04 for the 3-, 4-, and 5-mm rods, respectively), with values similar to those acquired using a single mouse bed (1.05 ± 0.11, 1.04 ± 0.05, and 1.02 ± 0.04, respectively; Fig. 2B). Interestingly, when imaging a single phantom in the mouse hotel, with the exception of the 3-mm rod, which was placed farthest from the center of the field of view, all RCs were within tolerable limits at all rod diameters tested, presumably because of the presence of a single point-source of radioactivity (0.23 ± 0.02, 0.99 ± 0.09, 1.2 ± 0.08, 1.1 ± 0.08, and 1.1 ± 0.03 for 1-, 2-, 3-, 4-, and 5-mm rods, respectively).

The SORs for both the water-filled and the air-filled chambers were within the tolerable limit of 15% for all imaging configurations, suggesting that an appropriate scatter correction was applied. SORs for the mouse hotel were significantly higher than those for a single mouse bed, however. For configurations 1, 2, and 3, the water SOR was 9.7% ± 0.7%, 6.2% ± 0.8%, and 4.9% ± 0.8%, respectively (*P* = 0.0003 and *P* < 0.0001 for configuration 1 vs. 2 and configuration 1 vs. 3, respectively). The air SOR was 7.8% ± 0.08%, 4.9% ± 0.4%, 4.5% ± 0.4% for configurations 1, 2, and 3, respectively (*P* = 0.0001 and *P* < 0.0001 for configuration 1 vs. 2 and configuration 1 vs. 3, respectively; Fig. 2C). The variation in activity concentration, as measured by the percentage SD in the uniform region of the phantom, was higher when 4 phantoms were imaged simultaneously than when the single-phantom configurations were used, representing elevated image noise (3.4% ± 0.35%, 2.3% ± 0.27%, and 2.2% ± 0.1% for configurations 1, 2, and 3, respectively; *P* = 0.003 for configuration 1 vs. 2; *P* = 0.004 for configuration 1 vs. 3; Fig. 2D). All values, however, were well below the tolerable limit of 15%. Representative single-slice ^18^F-FDG PET/CT images (0- to 20-min summed activity) of the phantoms are displayed in Figure 2E, comparing the 3 imaging configurations.

### Physiologic Regulation

**FIGURE 3.**
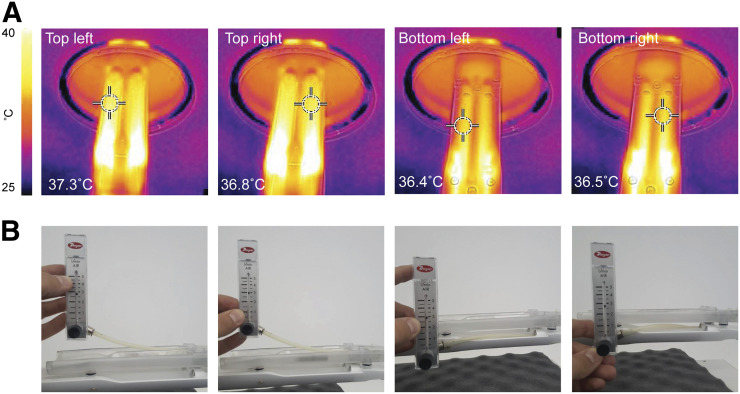
Testing of temperature and anesthesia flow rate uniformity in mouse hotel. (A) Temperature results for the 4 positions. (B) Flowmeter values representing anesthesia output from each nose cone.

The mouse hotel was designed to circulate heated air evenly across the 4 beds to facilitate a constant and even temperature distribution (36.8°C ± 0.4°C; Fig. 3A). As a design consideration, air inlets, where temperatures higher than 37°C were observed, were placed toward the back of the bed, away from the region of the bed where the mice were positioned. Flowmeter readings showed that anesthesia was distributed evenly to each nose cone (2.8 ± 0.1 L/min; Fig. 3B).

### Animal Studies

**FIGURE 4.**
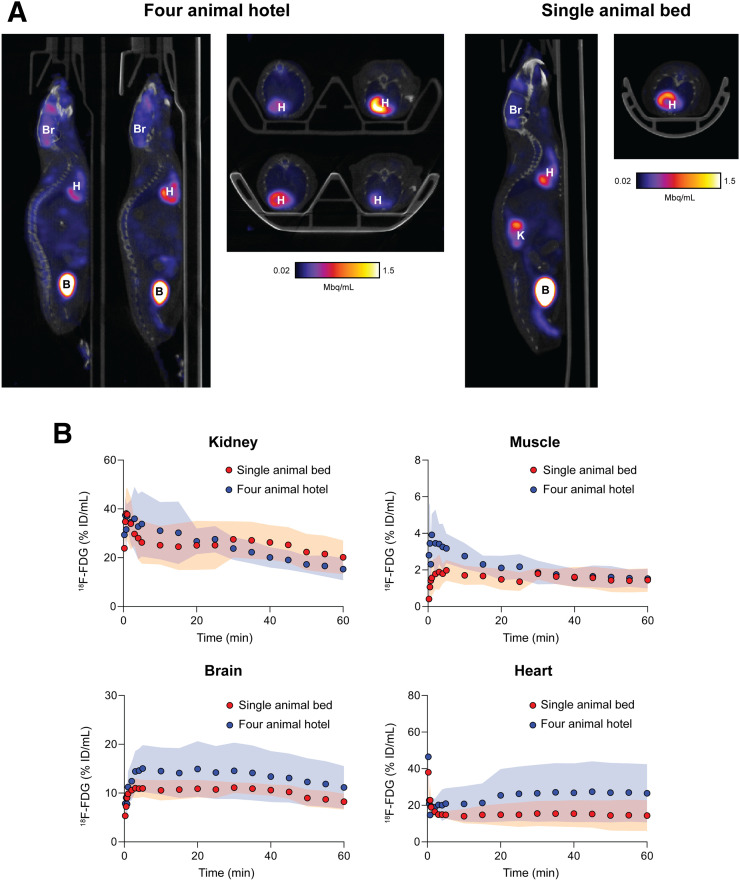
(A) Representative sagittal and axial PET images of mice 30–60 min after injection of tracer. B = bladder; Br = brain; H = heart; K = kidney. (B) Time–activity curves for major organs of interest normalized to percentage injected dose (ID). Shaded regions represent 1 SD from mean.

To evaluate the mouse hotel under standard in vivo imaging conditions, 4 healthy mice were positioned on the bed and simultaneously imaged with ^18^F-FDG PET dynamically over 60 min (Fig. 4A). An additional 4 mice were imaged in a single mouse bed for comparison. After reconstruction, time–activity curves revealed low levels of variability in ^18^F-FDG uptake for all major organs for animals imaged in the mouse hotel (Fig. 4B). There was no significant difference in the area under the time–activity curve for any major organs (Supplemental Fig. 2). When compared with the single-mouse-bed scans, however, the variation in radiotracer tissue uptake between subjects increased with the mouse hotel. Representative maximum-intensity projections after manual removal of the bed are shown in Supplemental Figure 3.

## DISCUSSION

Here, we sought to validate the performance of a 4-mouse imaging bed for use in a nanoScan PET/CT system. The use of a mouse hotel has a major benefit over conventional single-mouse-bed imaging, saving investigators time and reducing overall costs. Additionally, by increasing the number of animals that can be scanned for the same cost and time, the statistical power and corresponding confidence in the imaging data can be substantially increased.

The feasibility of simultaneously imaging more than one animal in the same PET/CT system has been reported for multiple studies (8–10,12), facilitated through the development of new small-animal PET/CT platforms that offer sensitive and reproducible imaging over both a large axial and a large transaxial field of view. To the best of our knowledge, however, physiologic regulation and monitoring have not been incorporated into the user-developed animal holders currently in existence. Unlike previous unheated animal bed holders, which can be used for only a short duration (12), dynamic imaging over multiple hours is achievable with the mouse hotel as a result of fine temperature regulation. Changes in environmental conditions can affect mammalian physiology, including disruption of thermoregulation, respiration, and cardiac output (13). Maintaining a constant bed temperature will therefore limit variations in radiotracer pharmacokinetics while minimizing any potential pain, suffering, distress, or lasting harm to the animal.

To evaluate the performance of the mouse hotel, phantom tests were performed under the conditions of the NEMA NU-4 2008 standard protocol for small-animal PET systems and using the reconstruction parameters suggested by the manufacturer (Supplemental Fig. 1). To improve on the suggested parameters for a single mouse bed, we increased the number of iterations from 4 to 10 and found low-level radioactivity in the water and air chambers due to photon scattering. The SOR in both the air and the water chambers was increased for the mouse hotel compared with the single-mouse-bed images, as can be attributed to the increased number of subjects in the field of view, leading to elevated photon scattering. The amount of scatter in the nonradioactive chambers using the mouse hotel, however, was below the maximum tolerable limit recommended by the manufacturer, suggesting an acceptable decrease in image quality.

To assess the expected impact on spatial resolution in the mouse hotel, RCs were calculated for the 1- to 5-mm rods in the phantom. For the 1- and 2-mm rods, the RCs were decreased by 76% and 26%, respectively, compared with phantoms scanned on a single mouse bed. For the larger-diameter rods, all values fell within tolerable limits. A reduction in spatial resolution is expected with the mouse hotel because each imaging chamber is placed away from the center of the field of view. As previously found (14), increasing the number of iterations from 4 to 10 during reconstruction improved the RCs of all rods, but image noise was amplified and, subsequently, observable image quality was reduced. During small-animal PET imaging of tumor-bearing mice, lowered spatial resolution is unlikely to alter quantitative information because most tumors imaged are typically larger than 50 mm^3^; further optimizing the number of iterations during reconstruction may therefore not be beneficial. Care, however, should be taken when evaluating radiotracer uptake in small tissues, such as lymph nodes or small metastatic lesions, when using the mouse hotel.

Systematic bias may be introduced through possible differences in scanner performance at each of the 4 positions of the mouse hotel. To address this potential confounder, we assessed quantitative accuracy and precision using a single phantom and corresponding NEMA tests at each of the 4 bed positions (Figs. 2B–2E). Minimal differences in RCs, SORs, and uniformity were found between all 4 bed positions, as shown by the small SD of the observed measurements. This result was encouraging, as variation in performance at the different bed positions was taken into consideration at the design stage, whereby all positions were set to be equidistant from the center of the field of view (Fig. 1B). In vivo, ^18^F-FDG radiotracer uptake in the major organs of healthy mice scanned simultaneously versus those scanned individually produced similar quantitative values, despite the expected biologic variation in radiotracer retention and excretion. To ensure maximum image quality, applying scatter and attenuation correction to all in vivo imaging studies using the mouse hotel is highly recommended.

Although we have demonstrated the feasibility of simultaneous imaging of up to 4 mice while maintaining quantitative precision, there are several improvements in bed design that should be incorporated before commercialization. At the time of data acquisition, both breathing and heart rate monitoring were available for only 1 of the 4 mice using this prototype bed. An updated bed that incorporates physiologic monitoring for all 4 mice has subsequently been developed. An additional consideration is the potential degradation of image quality when 4 animals are imaged simultaneously. Here, phantom imaging studies revealed potential issues with image reconstruction, leading to loss of data quality. Suboptimal image reconstruction and lowered image resolution were initially seen through the loss of detected radioactivity in the small 1- and 2-mm rods of phantoms scanned in the mouse hotel. Furthermore, in our in vivo studies, increased variation in ^18^F-FDG uptake in both the brain and the heart was observed in the time–activity curves of animals scanned using the mouse hotel versus a single mouse bed (Fig. 4B). Although this variation might be biologic, due to alterations in cardiac output and metabolism between subjects, for example, caution should be applied when analyzing image-derived outputs using small numbers of animals. Future work will assess bed performance in higher-powered studies with a range of different radiotracers.

Despite some minor drawbacks, a considerable benefit of using the mouse hotel during PET imaging is the large cost savings compared with a conventional single-mouse-bed scanner. A clinical dose of approximately 370 MBq of ^18^F-FDG costs approximately £300 ($369) in the United Kingdom. Considering the time required to position the animal on the bed, to prepare the radioactive dose, and to run a 10-min CT scan, with a constantly decaying radiotracer, each clinical dose will usually allow for approximately five 60-min dynamic scans. For novel radiotracer development and discovery in PET imaging, for which the radioactivity concentration received may be substantially less than a clinical dose of ^18^F-FDG, the number of consecutive scans that can be achieved is even fewer. For sufficiently powered studies, a sample size of 8 or more animals is typically required to identify moderate changes in radioactive concentration with statistical confidence. Performing 60-min dynamic ^18^F-FDG PET/CT on a conventional single mouse bed would require a minimum of 2 radioactive preparations to achieve this statistical power. Additionally, imaging institutes may charge approximately £200 ($246) per hour for use of the scanner. To image 8 mice using a single mouse bed, a total of 12 h split over 2 d would be required. However, imaging 8 mice using the mouse hotel would require a maximum of only 4 h in 1 d. In total, we estimate that scanning and radiotracer costs for the mouse hotel could be reduced by approximately 60% using commercially available ^18^F-FDG, with far higher savings potentially achieved using low-yielding novel radiotracers.

## CONCLUSION

A mouse hotel was designed to help imaging scientists perform more time-efficient, cost-effective research by quadrupling the number of mice that can be imaged in a single session. In particular, in experiments using short-lived isotopes, multiple animals can be scanned using a single synthesis, which would otherwise not be possible. The design of the mouse hotel allows for uniform control over temperature and anesthesia, with phantom studies and in vivo imaging of mice confirming its utility in increasing the throughput of PET imaging without considerable loss of image quality or quantitative precision. In comparison to a single mouse bed, the cost and time associated with each scan were substantially reduced.

## DISCLOSURE

This study was funded through a Wellcome Trust and Royal Society Sir Henry Dale Fellowship (107610/Z/15/Z) and a CRUK UCL Centre Non-Clinical Training Award (A23233) to Timothy H. Witney. Gábor Mócsai, Zoltán Nyitrai, and Sandor Hobor are employed by Mediso Medical Imaging Ltd. but do not own any shares or have any financial benefits other than general compensation for employment. They do not hold any patents related to the topic of the publication. No other potential conflict of interest relevant to this article was reported.

## KEY POINTS

**QUESTION:** Can a mouse hotel that was developed to simultaneously image up to 4 mice reduce costs and maximize the efficiency of radiotracer use when compared with scans performed with a single mouse bed?
**PERTINENT FINDINGS:** Analysis of images acquired using the mouse hotel confirmed its utility to increase the throughput of small-animal PET imaging without considerable loss of image quality or quantitative precision, with the cost and time associated with each scan being substantially reduced.
**IMPLICATIONS FOR PATIENT CARE:** Small-animal imaging experiments are vital to the successful development of novel radiotracers and are required for biologic validation of these tracers before their translation to the clinic. By increasing the throughput of preclinical imaging 4-fold, the mouse hotel allows high-powered studies to be completed cheaper and faster than conventional scanning using a single mouse bed.
